# TFIIB-related factor 2 inhibits lung squamous carcinoma cell apoptosis through *SLC8A3*-mediated mitochondrial homeostasis

**DOI:** 10.1038/s41419-025-07813-8

**Published:** 2025-07-03

**Authors:** Shen Yi, Xing Qi, Fei Luo, Dingxin Wang, Zitong Feng, Luyuan Ma, Wenhao Yu, Cong Wang, Hui Tian, Ming Lu

**Affiliations:** 1 Department of Thoracic Surgery, Qilu Hospital, Cheeloo College of Medicine, Shandong University, Jinan, China; 2 School of Public Health, North China University of Science and Technology, Tangshan, China; 3 Department of Thoracic Surgery, The First Affiliated Hospital of Shandong First Medical University and Shandong Provincial Qianfoshan Hospital, Jinan, China; 4 Department of Radiation Oncology, Qilu Hospital, Cheeloo College of Medicine, Shandong University, Jinan, China

**Keywords:** Oncogenes, Translational research

## Abstract

Lung cancer is the most common cancer and the leading cause of cancer-related deaths. Developing therapies for lung cancer is challenging, and new targets are urgently required. TFIIB-related factor 2 (BRF2) plays a crucial role in the development and progression of various tumors. However, the potential role of BRF2 in lung squamous carcinoma (LUSC) is unclear. Therefore, the aim of this study was to elucidate the mechanism of BRF2 regulation in LUSC development. Flow cytometry, protein blotting, and in vivo experiments were performed to assess the function of BRF2 in LUSC. Transmission electron microscopy imaging and mitochondrial membrane potential (MMP) measurements were used to determine the effect of BRF2 on mitochondria in LUSC. The impact of the downstream molecule SLC8A3 was predicted using bioinformatics analysis, and the mechanism was investigated by analyzing quantitative reverse transcription-polymerase chain reaction and immunoprecipitation (IP) assays, which were confirmed through rescue experiments. BRF2 expression was upregulated in squamous carcinoma cells, which increased SLC8A3 protein expression, promoted mitochondrial autophagy, stabilized MMP, and reduced apoptosis. In addition, *SLC8A3* overexpression inhibited PTEN-induced putative kinase 1 (PINK1) binding to TIMM23 to promote mitochondrial autophagy and stabilize the MMP, which counteracted BRF2 knockdown-induced apoptosis. BRF2 mediated *SLC8A3* expression to reduce apoptosis in LUSC cells by maintaining mitochondrial homeostasis. These findings provide novel selective therapeutic targets and ideas for the treatment of LUSC.

## Introduction

Lung cancer is the most common cancer, accounting for approximately 2.5 million new cases in 2022, and one-eighth of all cancers worldwide [1]. Lung cancer is also the leading cause of cancer-related deaths and has a considerably higher mortality rate than other cancers, despite the tremendous progress made recently in its diagnosis and treatment through surgery, targeted therapy, and immunotherapy and the decreased lung cancer mortality rates [2, 3]. Non-small cell lung cancer (NSCLC) accounts for 85% of lung tumors, and lung squamous cell carcinoma (LUSC), a subtype of NSCLC, accounts for 20–30% of all NSCLC cases [4, 5]. However, despite recurrent molecular aberrations in LUSC, developing targeted therapies against receptor tyrosine kinases, signaling pathways, and cell cycle checkpoints has been considerably challenging [6]. Therapies using LUSC-specific metabolic vulnerabilities, such as those targeting gluconeogenesis, have also been proposed [7, 8]. These novel therapies may act synergistically with immune checkpoint inhibitors. Therefore, discovering novel metabolic targets is crucial for diagnosing and treating LUSC.

TFIIB-related factor 2 (BRF2) is a member of the TFIIB-like core transcription family [9]. BRF2 recruits RNA polymerase III to the external promoter of type III genes and transcribes small untranslated RNAs involved in essential metabolic processes such as mRNA processing and translation [10, 11]. The capacity for redox sensing has also been reported, with its overexpression protecting cells from oxidative stress-induced apoptosis [12]. BRF2 is overexpressed in many malignant tumors and plays a crucial role in carcinogenesis [13–15]. Our previous study found that the overexpression level of BRF2 could act as a significant prognosis predictor for LUSC [16]. However, the role of BRF2 in LUSC and the mechanism by which it controls oxidative stress remain largely unknown. Therefore, the aim of this study was to elucidate the mechanism of BRF2 regulation in LUSC development.

## Materials and methods

### Cell culture and processing

The human respiratory epithelial cell lines (HBE135-E6E7, BEAS-2B), human embryonic kidney 293 T cells (HEK293FT), and human lung squamous cell carcinoma cell line (NCI-H520, NCI-H226) were purchased from the Shanghai Cell Bank Research Center, Chinese Academy of Sciences. The cell lines were grown in RPMI-1640 (Gibco, Waltham, MA, USA) and Dulbecco’s modified Eagle medium (Gibco) in a humidified incubator containing 5% CO_2_ at 37 °C. All media were supplemented with 10% fetal bovine serum (Gibco). Cells were certified for analysis every 3–6 months using short tandem repeat sequences and were monitored every 3 months using the MycoAlert Mycoplasma Detection Kit (Lonza). The H520 and H226 cell lines were certified on May 15, 2023, by Qingke Biotechnology (Beijing, China). Experiments were performed one week after cell thawing. For replicate experiments, cells were cultured for a maximum of two months. Negative control (NC), BRF2 and TIMM23 small interfering RNA (siRNA), slc8a3, TIMM23, and PTEN-induced putative kinase 1 (PINK1) overexpression plasmids were provided by Keyybio (Shandong, China) and GenePharma (Shanghai, China)(Supplementary Table S1). BRF2 lentivirus was supplied by Genechem (Shanghai, China). Cells were transiently transfected with validated siRNA and with the corresponding negative control siRNA using jetPRIME Transfection Reagent (Polyplus, Illkirch-Graffenstaden, France) according to the manufacturer’s instructions. LUSC cells were transfected with lentivirus and the corresponding negative control to construct a stable transformation model. Experiments were performed 48–72 h after transfection.

### IHC analysis

LUSC tissues were fixed with 4% paraformaldehyde. Following incubation at 60 °C for 1 h, dewaxing, and rehydration of the samples, antigen retrieval was performed using citrate buffer at 97 °C for 20 min. Peroxidase blocker was applied at room temperature for 10 min. After blocking with 5% normal goat serum in PBST at 37 °C for 2 h, the slides were incubated overnight at 4 °C with the BRF2 primary antibody. Each slide was then treated with an HRP-conjugated secondary antibody, washed three times with TBST, and developed using a DAB solution prior to counterstaining with hematoxylin. Images were captured using an inverted microscope. This study was conducted in accordance with the Declaration of Helsinki, with the ethical approval of the Medical Ethics Committee of Qilu Hospital of Shandong University (Coe). Patients were willing to participate and signed informed consent.

### Western blotting

Cells and tissue samples were lysed in RIPA buffer (Beyotime, Jiangsu, China) to extract proteins. Proteins were separated using 10% or 12% sodium dodecyl-sulfate polyacrylamide gel electrophoresis gels and then electrotransferred to polyvinylidene difluoride membranes, which were blocked with 5% skimmed milk for 1 h to prevent nonspecific antibody binding and then treated with primary antibodies (Supplementary Table S2) overnight at 4 °C. After incubation with the appropriate secondary antibody, the proteins were detected using an enhanced chemiluminescence system.

### Immunoprecipitation assay

Human embryonic kidney 293 T cells were lysed in an immunoprecipitation (IP) buffer (P0013; Beyotime); then, 500 µl protein lysate protein was removed from them as a control motif, and 500 µl protein lysate was mixed with 2 µg of antibody against FLAG and IgG by spinning at 4 °C for 1 h. Afterwards, 30 μL of protein A/G plus agarose was added to the mixture, which was then incubated overnight at 4 °C on a rotator. The following day, the mixture was mixed five times with IP buffer, and the precipitated proteins were mixed with the upsampling buffer for protein blotting analysis. Finally, protein blotting was performed to detect TIMM23 and PINK1 expression.

### Transcriptome sequencing, protein characterization by mass spectrometry, and bioinformatics analysis

LC Bio-Technology (Shanghai, China) performed transcriptome sequencing. The mRNA transcripts were identified by sequencing the transcriptome and were expressed differently in BRF2 knockdown H520 cells than in control H520 cells. Kyoto Encyclopedia of Genes and Genomes (KEGG) pathway enrichment map and Gene Ontology (GO) enrichment map were mapped based on the Sangerbox 3.0 platform (http://sangerbox.com).

### Transmission electron microscopy imaging

The relevant siRNAs transfected into H520 and H226 cells were digested using trypsin, collected by centrifugation (161×*g*), resuspended in 0.5% glutaraldehyde fixative for 10 min, centrifuged again (13500×*g*), and fixed with 3% glutaraldehyde. Finally, the cell samples were refixed with 1% osmium tetroxide, progressively dehydrated in acetone, and embedded in Ep812. The samples were then stained and cut using a diamond knife. The sections were examined using a transmission electron microscope (JEOL, JEM-1400-FLASH, Japan).

### Quantitative reverse transcription polymerase chain reaction

Total RNA was isolated from LUSD and tumor cells using the TRIzol reagent (Invitrogen, Waltham, MA, USA). cDNA was reverse-transcribed from RNA to cDNA using a reverse transcription kit (Aikerui Biotechnology, Hangzhou, China). Quantitative real-time PCR was performed on a Bio-Rad CFX Connect (Bio-Rad Laboratories, Hercules, CA, USA) using SYBR Premix Ex Taq (Aikerui Biotechnology, Hangzhou, China) with three sub-well replicates. Thermocycling conditions were chosen according to the manufacturer’s protocol. Relative gene expression was then analyzed using the ΔΔCq method. Each experiment was performed at least three times.

### Flow cytometry analysis

Apoptosis was analyzed using the Membrane-Linked Protein V-FITC/PI Apoptosis Detection Kit (Vazyme, Nanjing, China). Cells were collected 48 h after transfection, washed twice with phosphate-buffered saline (PBS), and suspended in 500 μL binding buffer. Subsequently, 5 μL of membrane-bound protein V-FITC and 5 μL of propidium iodide (PI) were added to the cell suspension. Cells were incubated in the dark at 15–25 °C for 15 min, and late and early apoptosis rates were measured using flow cytometry (CytoFLEX Beckman Coulter, Pasadena, California, USA).

### Mitochondrial membrane potential measurements

Mitochondrial membrane potential (MMP) changes were measured using a Mitochondrial Membrane Potential Assay Kit with JC-1 (Beyotime) according to the manufacturer’s instructions. Briefly, treated cells were harvested and washed twice with cold PBS. After that, the cells were resuspended in a mixture of 500 μL of culture medium and 500 μL of JC-1 staining solution for 20 min at 37 °C in the dark. The cells were then washed thrice with cold staining buffer before flow cytometry (Olympus, Tokyo, Japan). JC-1 is present as a cytoplasmic JC-1 monomer or as a mitochondrial J aggregate, depending on the MMP. In healthy cells with a high MMP, JC-1 spontaneously forms J-aggregates in the mitochondria and emits red fluorescence. However, in unhealthy cells, where MMP is decreased, JC-1 is released from the mitochondria and exists as a monomer in the cytoplasm, resulting in green fluorescence. Therefore, MMP can be expressed as the ratio of red-to-green fluorescence intensity. MMP changes were detected in situ using the JC-1 assay. After appropriate treatment, the cells were stained with JC-1 as described above and visualized using laser scanning confocal microscopy (Nikon, Japan).

### Membrane-associated protein V/PI staining

The mode of cell death was determined using a Membrane Linker V-FITC Apoptosis Detection Kit (KeyGen Biotech, Nanjing, China). Briefly, MCs were harvested using trypsin digestion after treatment, rinsed twice with cold PBS, and resuspended in 500 μL of binding buffer. The cells were then stained with 5 μL of Annexin V-FITC and 5 μL of PI for 15 min at room temperature in the dark. Stained MCs were analyzed using flow cytometry (Olympus).

### In vivo experiments

The Medical Ethics Committee of Qilu Hospital of Shandong University approved and supervised all animal experiments. Animal experiments follow ARRIVE guidelines. Four-week-old Female BALB/c nude mice were obtained from Beijing Vital River Laboratory Animal Technology (Beijing, China). Transduced H520 and H226 cells were injected subcutaneously into the right axillary region of each mouse. Each cell line was divided into a negative control group and a BRF2 knockdown group, forming a total of four groups. In the rescue experiment, SLC8A3 overexpression plasmid and blank vector were transfected on the basis of the original stable cells. The animals were randomly divided into different groups using a double-blind approach to minimize experimental bias. The tumor size was measured every 5 days. Tumor volume was calculated using the following formula: Volume = (length × width × width)/2. After 15 d, these mice were euthanized via exposure to carbon dioxide gas in an uncrowded tank. The tumors were collected and weighed. Mouse tumor tissues were fixed with 4% paraformaldehyde. The tumor samples were embedded in paraffin and sectioned. Terminal deoxynucleotidyl transferase dUTP nick end labeling (TUNEL) staining was used to assess tissue apoptosis using a Fluorescein (FITC) TUNEL Cell Apoptosis Detection Kit (Servicebio, Wuhan, China) according to the manufacturer’s instructions.

### Statistical analysis

Statistical analyses were performed using GraphPad Prism 8.0 (GraphPad Software, San Diego, CA, USA) and IBM SPSS Statistics 25 (SPSS Inc., Chicago, IL, USA). Data are presented as the mean ± standard deviation. The *t*-test was used to analyze the differences between the two groups, and the chi-square test was used to analyze the categorical variables. Statistical significance was set at *P* < 0.05.

## Results

### *BRF2* expression was upregulated in LUSC tissues and cells

**Fig. 1:**
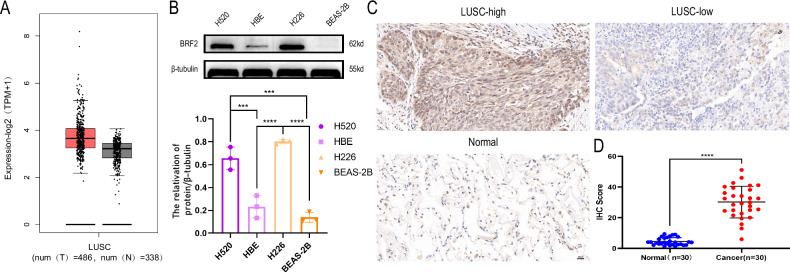
TFIIB-related factor 2 (BRF2) was highly expressed in lung squamous cell carcinoma tissues and lung cancer cell lines. **A** *BRF2* mRNA expression level in lung squamous carcinoma was higher than in normal tissues according to the Cancer Genome Atlas dataset. Red: tumor; black: normal; and ***P < 0.001. **B** BRF2 expression in LUSC cell lines (H520 and H226) was higher than in human normal bronchial epithelial cells (HBE) and human normal lung epithelial cells (BEAS-2B). **C** Representative IHC images demonstrating the expression level of BRF2 in LUSC tissues and normal tissues. **D** The IHC scores of BRF2 in LUSC tissues were higher than normal lung tissues (****P < 0.0001).

According to the bioinformatics analysis of several databases, BRF2 expression was significantly higher in LUSC tissues than in normal lung tissues (Fig. 1A). Western blotting revealed that BRF2 was highly expressed in the H520 and H226 cell lines (Fig. 1B). The results showed that BRF2 was highly expressed in lung squamous cell carcinoma tissues and cells. IHC staining was performed to determine the expression of BRF2 protein in cancer tissues and adjacent non-cancerous tissues of 30 LUSC patients (Fig. 1C). The IHC scores revealed that the expression of BRF2 was upregulated in LUSC tissues (Fig. 1D).

### BRF2 silencing increased LUSC cell apoptosis in vitro and in vivo

**Fig. 2:**
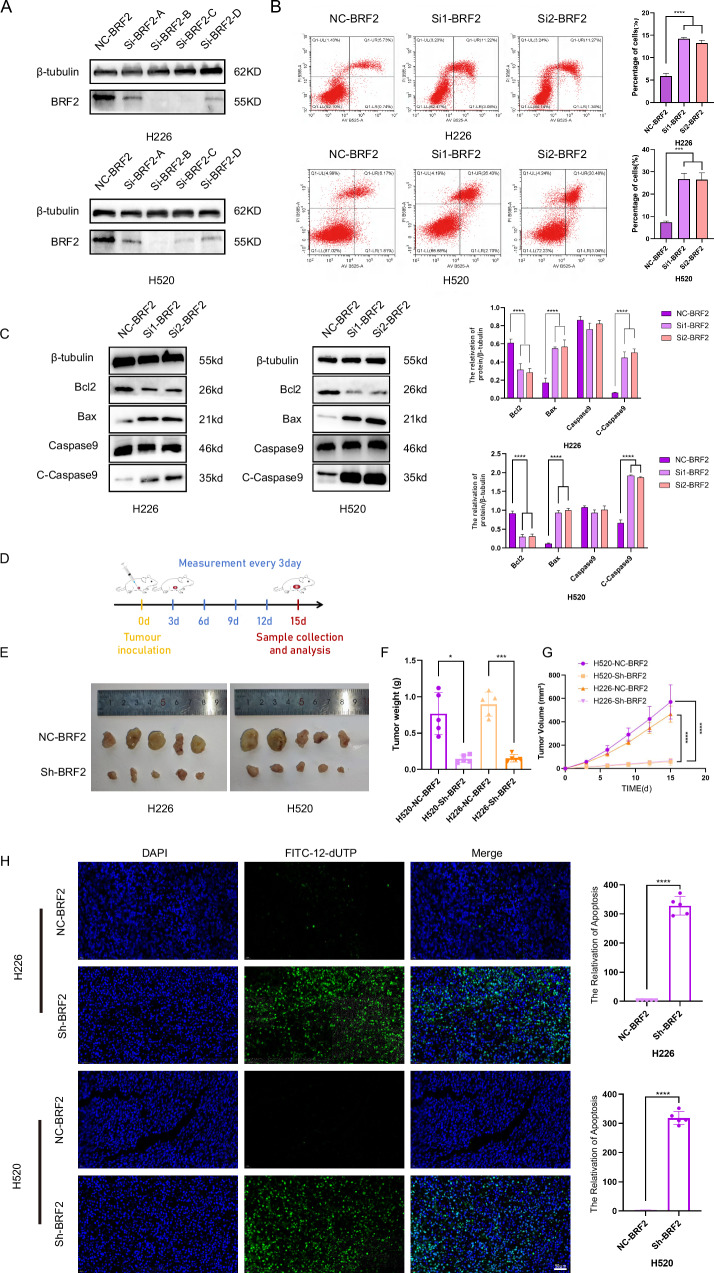
BRF2 silencing increased LUSC cell apoptosis in vitro and in vivo. **A** Western blot analysis revealed that sequences B and C exhibited superior interference efficacy. **B** BRF2 knockdown increased apoptosis in H520 and H226 cells (**P < 0.01; ***P < 0.001). **C** Compared to the control group, the Bax and cleaved caspase-9 protein levels increased, whereas the BCL-2 protein level decreased in BRF2 knockdown groups. Protein levels are expressed as the mean ± standard deviation (SD); *n* = 3. ****P < 0.0001. **D** H520 and H226 cells were subcutaneously implanted into the right axillary region of 4-week-old nude BALB/c mice. The tumor size was measured every 3 days. On day 15, each tumor was collected and weighed. **E** Representative images of tumors in nude mice after tumor injection. The tumor mass in the BRF2 knockdown group was smaller than that in the control group. **F** The tumor weight of the BRF2 knockdown group was lower than that of the control group. **G** The tumor volume of the BRF2 knockdown group was smaller than that of the control group. **H** Tumor tissue sections were labeled with 4′,6-diamidino-2-phenylindole (DAPI), and FITC-12-dUTP, and cell apoptosis was observed using confocal microscopy. The BRF2 knockdown group showed higher TUNEL positivity than the control group. Left: Representative micrographs; right: TUNEL-positive rate statistics, *n* = 5. DAPI, blue; FITC-12-dUTP, green. Scale bar: 20 µm. (***P < 0.001; ****P < 0.0001).

After verifying the knockdown efficiency of four small interfering sequences in H226 and H520 cells, the two most efficient sequences, Si1-BRF-B and Si2-BRF-C (Fig. 2A), were selected to characterize BRF2 expression in LUSC cells. After transfection of the two LUSC cell lines with small interfering sequences, flow cytometric analysis using Annexin V/PI staining showed that BRF2 knockdown increased LUSC cell apoptosis (Fig. 2B). BRF2 knockdown in H520 and H226 cells increased Bax and cleaved caspase-9 protein expression, whereas decreasing BCL-2 protein expression compared to the control group. These results suggest that BRF2 reduces apoptosis in LUSC cells (Fig. 2C). BRF2 stable knockdown using lentivirus in H520 and H226 cell lines was implanted in nude mice to establish a tumorigenesis model (Fig. 2D). BRF2 knockdown significantly reduced the tumor growth rate, volume, and weight in nude mice (Fig. 2E–G). TUNEL staining confirmed tumor cell apoptosis in nude mice, and compared to the control group, BRF2 knockdown was associated with an increased level of tumor cell apoptosis (Fig. 2H). These results suggest that BRF2 knockdown increases LUSC cell apoptosis in vitro and in vivo.

### BRF2 affected mitochondrial function in LUSC cells

**Fig. 3:**
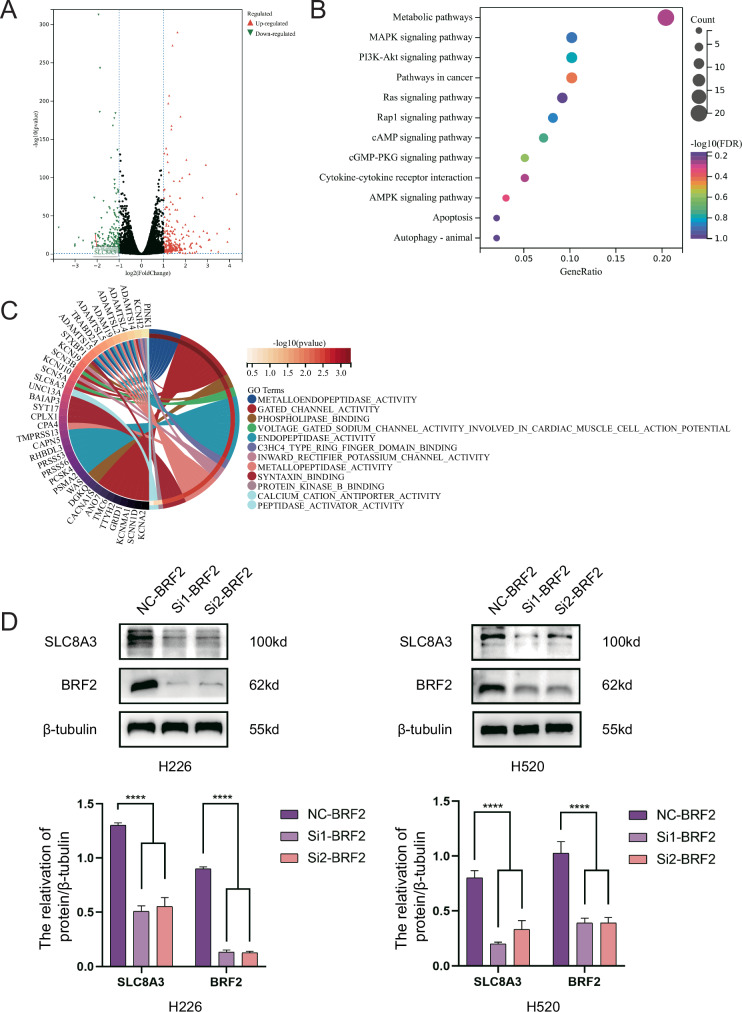
BRF2 affected mitochondrial function in LUSC cells. **A** BRF2 variance analysis of single-genes differentially expressed a volcanic figure according to The Cancer Genome Atlas LUSC database. Fold change ≥ 1; *P* < 0.05. **B** Downregulated genes in differential expression analysis correspond to Kyoto Encyclopedia of Genes and Genomes pathway enrichment profiles. **C** Circle plot of enrichment analysis of Gene Ontology entries corresponding to downregulated genes in the differential expression analysis. **D** SLC8A3 protein expression was downregulated in BRF2 knockdown cells. Protein levels are expressed as the mean ± SD; *n* = 3. ****P < 0.0001.

RNA sequencing of LUSC cells after BRF2 knockdown was performed to explore the molecular mechanisms underlying the effects of BRF2 on LUSC cells. A total of 604 differentially expressed genes (DEGs) (multiple change ≥ 1, *P* < 0.05) were identified between cells in the LUSC-NC control and LUSC-SiBRF2 groups (Fig. 3A). In total, 269 upregulated and 335 downregulated DEGs were identified. KEGG analysis indicated that pathways in cancer, metabolism, AMPK signaling, apoptosis, autophagy, animal, and cytokine–cytokine receptors were strongly correlated among the top 10 signaling pathways enriched with downregulated DEGs in H520 cells (false discovery rate < 0.05) (Fig. 3B).

The downregulated genes were further screened to identify the downstream molecules regulated by BRF2 (fold change ≥ 2, *P* < 0.05), and 16 downregulated DEGs were obtained. After excluding genes most significantly associated with the biological process GATED_CHANNEL_ACTIVITY in Gene Ontology (GO) functional classification (Fig. 3C) and those unrelated to mitochondria, we identified SLC8A3. We furtherly detected the expression level of SLC8A3 after BRF2 knockdown. We found that SLC8A3 protein expression decreased as expected (Fig. 3D). These results suggest that BRF2 regulates mitochondrial function through SLC8A3.

### BRF2 promoted mitochondrial autophagy and stabilized the mitochondrial membrane potential

**Fig. 4:**
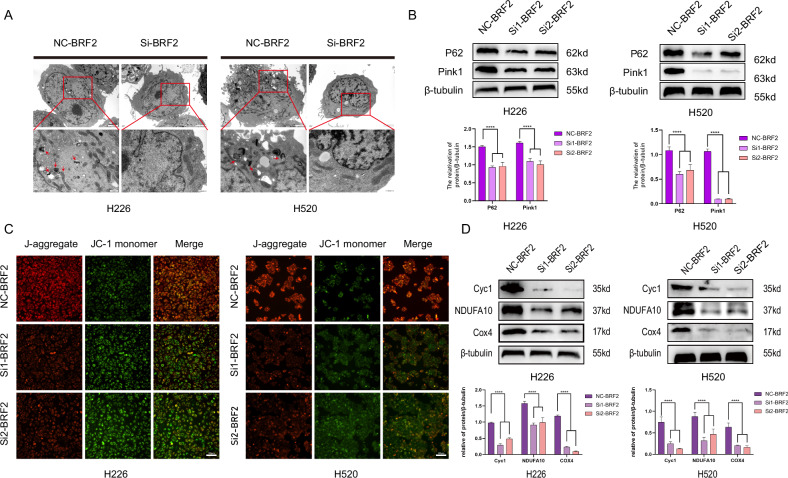
BRF2 promoted mitochondrial autophagy and stabilized the mitochondrial membrane potential. **A** Transmission electron microscopy of H520 and H226 cells. In the si-BRF2 group, mitochondrial structural damage (swelling, cristae disruption), double- or single-membrane structures enclosing mitochondria, autophagosome-lysosome fusion, and autophagosome numbers decreased compared to the control group. Experimental scale: 2 μm/500 nm. **B** Compared with the control group, the P62 and PINK1 protein levels were downregulated in BRF2 knockdown cells. Protein levels are represented as the mean ± SD; *n* = 3. ****P < 0.0001. **C** H520 and H226 cells were treated and labeled with the fluorescent probe JC-1, and mitochondrial membrane potential (MMP) changes in situ were observed using confocal microscopy. JC-1 forms J-aggregates and emits red fluorescence when the mitochondrial membrane potential is normal. JC-1 exists in monomeric form and emits green fluorescence when the membrane potential decreases. Representative micrographs showed that control cells showed higher levels of red fluorescence, whereas BRF2 knockout cells had higher levels of green fluorescence. J-aggregate: red; JC-1 monomer: green. Scale: 20 µm. **D** Compared to the control group, the protein levels of Cyc1, NDUFA4, and Cox4 were downregulated in the si-BRF2 groups. Protein levels are expressed as the mean ± SD; *n* = 3, ****P < 0.0001.

The cell status after BRF2 knockout in H520 and H226 cells was observed, and the expression of mitochondrial autophagy-associated proteins was detected after BRF2 knockout. In the si-BRF2 group, mitochondrial structural damage (swelling, cristae disruption), double- or single-membrane structures enclosing mitochondria, autophagosome-lysosome fusion, and autophagosome numbers decreased compared to the control group (Fig. 4A). Activation of PINK1 and Parkin leads to ubiquitination of mitochondrial outer membrane proteins, which is the initiation signal for mitochondrial autophagy [17]. p62 acts as an adaptor protein that recognizes these ubiquitination markers and connects the damaged mitochondria to the autophagosome. In this way, damaged mitochondria are efficiently encapsulated in the autophagosome and transported to the lysosome for degradation [18]. Thus, we furtherly detected the expression levels of PINK1 and P62 protein. BRF2 knockdown reduced PINK1 and P62 protein expression compared to control cells (Fig. 4B). These results suggest that BRF2 promotes mitochondrial autophagy. To further verify the status of mitochondria in LUSC cells, mitochondrial damage was verified (Fig. 4C), and the expression of proteins related to the mitochondrial oxidative respiratory chain was examined after BRF2 knockdown. BRF2 knockdown increased mitochondrial membrane depolarization and decreased Cyc1, NDUFA10, and Cox4 protein expression, which are the key proteins in the electron transport chain of mitochondria (Fig. 4D). These results suggest that BRF2 could promote mitochondrial autophagy and stabilize the mitochondrial membrane potential.

### *SLC8A3* attenuated PINK1 interaction with TIMM23

**Fig. 5:**
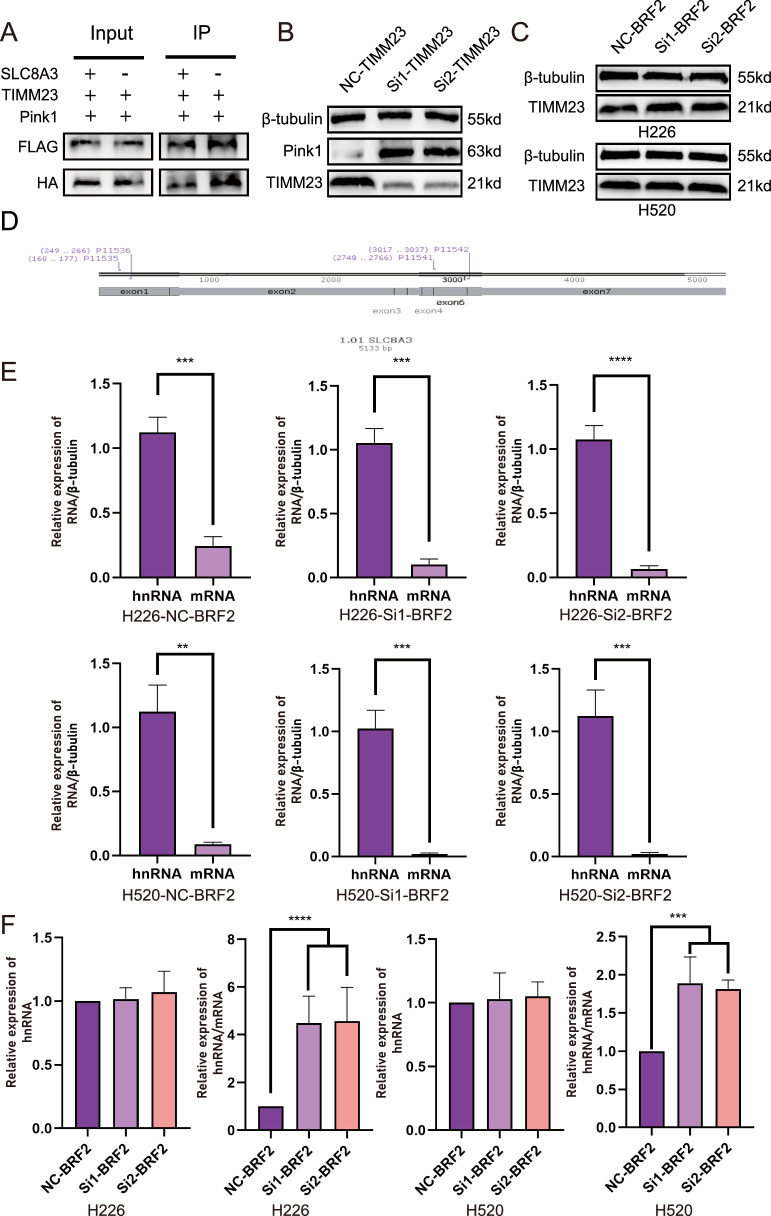
SLC8A3 attenuated PINK1 interaction with TIMM23 and BRF2 affected SLC8A3 expression by regulating its alternative splicing. **A** Exogenous immunoprecipitation confirmed that the interaction between TIMM23 and PINK1 was weakened by increased *SLC8A3* expression. **B** Compared with the control group, the PINK1 protein levels were downregulated in TIMM23 knockdown cells. **C** Compared with the control group, TIMM23 protein expression exhibited no significant changes in si-BRF2 groups. **D** mRNA *SLC8A3* protein primer on the position of primers 1 and 2; primer 1 in *SLC8A3* mRNA in exons 1 and 2, primers across *SLC8A3* mRNA exon 6 and 7. **E** qPCR detection of the H520 and H226 primer 1 and 2 reverse transcription products. Significant differences were observed in hnRNA and mRNA among the three transcribed groups. The *SLC8A3* protein level is considerably higher than the *SLC8A3* mRNA level. (*n* = 3. **P < 0.01; ***P < 0.001; ****P < 0.0001). **F** Results show that after BRF2 knockdown, the difference in *SLC8A3* transcribed the *SLC8A3* protein level and the protein mRNA level increased (*n* = 3. ***P < 0.001; ****P < 0.0001).

In addition to focusing on the expression level of PINK1, the strength of the interaction between PINK1 and mitochondrial inner membrane proteins is also a critical factor in regulating mitochondrial autophagy. TIMM23, as a core transport protein of the mitochondrial inner membrane, is involved in the recognition and transport of mitochondrially encoded proteins, and its function is closely related to PINK1-mediated mitochondrial autophagy [19, 20]. Based on this mechanism, we further investigated the impact of SLC8A3 on the interaction between PINK1 and TIMM23. Human embryonic kidney 293 T cells were transfected with the FLAG tag overexpression plasmid TIMM23 and the HA tag overexpression plasmid PINK1, and the existing system was transfected with the *SLC8A3* overexpression plasmid and the corresponding empty vector to verify whether *SLC8A3* can affect the interaction of PINK1 with TIMM23. The results showed that *SLC8A3* attenuated the interaction between PINK1 and TIMM23 (Fig. 5A). In TIMM23-knockdown human embryonic kidney 293 T (HEK293T) cells, undegraded PINK1 accumulated (Fig. 5B), whereas TIMM23 expression remained unchanged in lung squamous cell carcinoma cell lines H226 and H520 despite variations in BRF2 and SLC8A3 levels (Fig. 5C).

### BRF2 affected *SLC8A3* expression by regulating its alternative splicing

qPCR experiments were performed to further explore the mechanism through which BRF2 regulates *SLC8A3* expression. A sequence in exon 1 of *SLC8A3* was selected, and primer 1 was designed to represent the hnRNA of *SLC8A3*. Primer 2 was designed by selecting a sequence spanning between exons 6 and 7 to represent the *SLC8A3* mRNA (Fig. 5B) (Supplementary Table S2). Alternative *SLC8A3* splicing was observed in LUSC cells (Fig. 5C). The knockdown of BRF2 significantly reduces the abundance of mature SLC8A3 mRNA, whereas leaving the transcription levels of SLC8A3 hnRNA unaffected (Fig. 5D). These results suggest that BRF2 affected *SLC8A3* expression by regulating alternative splicing.

### *SLC8A3* overexpression antagonized the effect of BRF2 knockout on tumor cell apoptosis in vitro

**Fig. 6:**
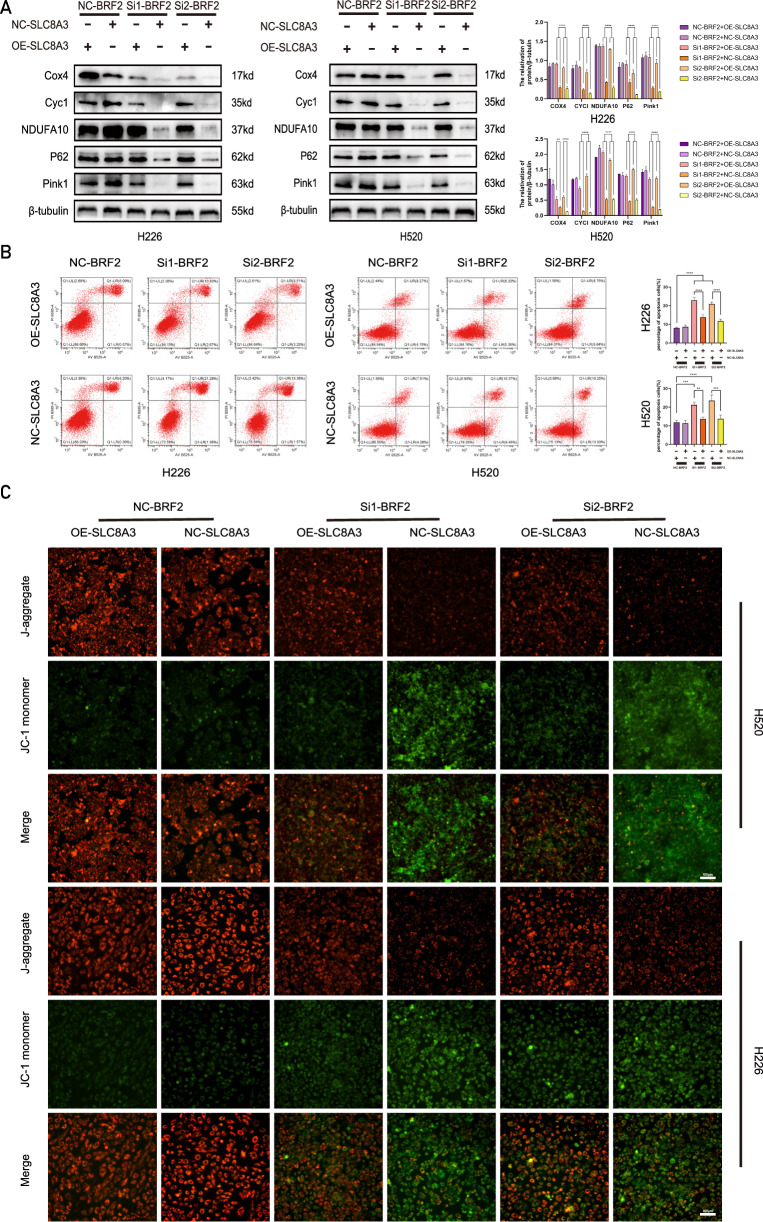
*SLC8A3* overexpression antagonized the effect of BRF2 knockdown on tumor cell apoptosis in vitro. **A** BRF2 knockdown reduced the expression levels of Cox4, Cyc1, NDUFA10, P62, and Pink1, whereas SLC8A3 overexpression counteracted the effects of BRF2 knockdown. Left: Representative western blotting data showing the levels of mitophagy- and respiratory chain-related proteins. Right: Protein levels are expressed as the mean ± SD; *n* = 3, ****P < 0.0001. **B** Flow cytometry was used to determine the effect of BRF2 on apoptosis. In BRF2 knockdown-treated cells, the apoptosis level was decreased, whereas the *SLC8A3* overexpression group reversed this trend (**P < 0.01; ***P < 0.001; ****P < 0.0001). **C** H520 and H226 cells were labeled with the fluorescent probe JC-1, and MMP changes were observed using confocal microscopy in situ. Representative micrographs show that the *SLC8A3* overexpression group had a higher level of red fluorescence, whereas the empty vector control group had a higher level of green fluorescence in BRF2 knockdown-treated cells. J-aggregates: red; JC-1 monomers: green. Scale bar: 20 µm.

BRF2 promoted mitochondrial autophagy and reduced mitochondrial damage, which influences tumor apoptosis. Therefore, rescue experiments were performed by overexpressing *SLC8A3* in BRF2 knockout cells to determine whether *SLC8A3* is involved in the effect of BRF2 on tumor apoptosis. Western blotting (Fig. 6A), immunofluorescence (Fig. 6B), and flow cytometry analysis (Fig. 6C) indicated that SLC8A3 overexpression significantly rescued MMP depolarization and apoptosis in BRF2 knockout cells. SLC8A3 overexpression rescued the changes in autophagy and respiratory chain-related proteins after BRF2 knockdown.

### *SLC8A3* overexpression antagonized the effect of BRF2 knockout on tumor cell apoptosis in vivo

**Fig. 7:**
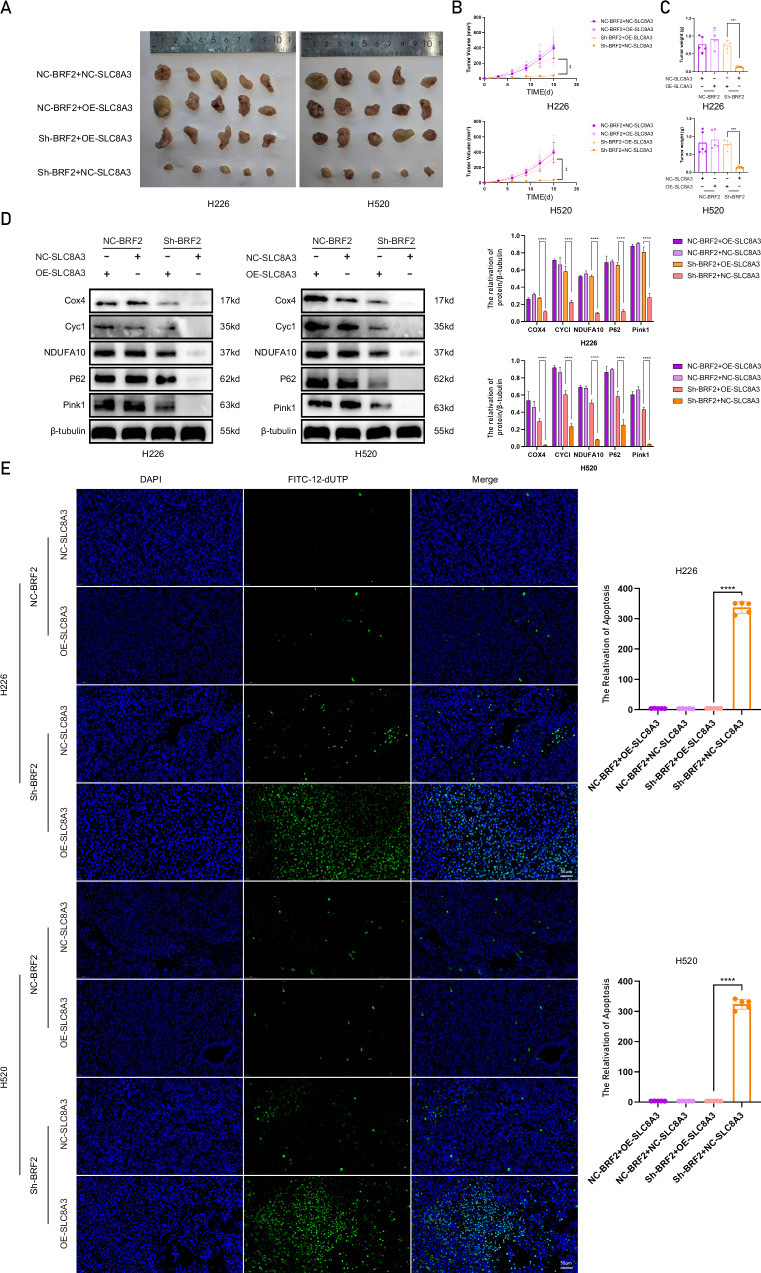
*SLC8A3* overexpression antagonized the effect of BRF2 knockout on tumor cell apoptosis in vivo. **A** Representative images of tumors in nude mice injected with H520 and H226 cells. The tumor growth rate was significantly suppressed in the BRF2 knockdown group compared to the control; however, this inhibitory effect was markedly reversed by SLC8A3 overexpression. **B**, **C** Overexpression of SLC8A3 effectively restored the impaired tumor weight and volume in BRF2 knockout nude mice (*n* = 5, *P < 0.05; ***P < 0.001). **D** Representative western blot data showing the levels of mitophagy- and respiratory chain-related proteins, such as Cox4, Cyc1, NOUFA10, P62, and Pink1. These protein expression levels decreased in the BRF2 knockdown group, whereas concomitant SLC8A3 overexpression rescued the inhibition in xenograft tumors. Target protein levels are expressed as the mean ± SD (*n* = 3. ****P < 0.0001). **E** Tumor tissue sections were labeled with DAPI and FITC-12-dUTP, and changes in apoptosis were observed using confocal microscopy. The BRF2 knockdown group exhibited an increased TUNEL-positive rate, whereas the SLC8A3 overexpression group reversed this trend. Left: representative micrographs, right: TUNEL positive rate statistics, *n* = 5, ****P < 0.0001. DAPI, blue; FITC-12-dUTP, green. Scale bar: 20 µm.

The in vivo rescue experiments demonstrated that overexpression of SLC8A3 effectively restored the impaired tumor growth rate, volume, and weight in BRF2 knockout nude mice (Fig. 7A–C). Western blot analysis further confirmed the rescuing effect of SLC8A3 expression, showing that BRF2 knockdown cells exhibited decreased expression levels of key mitophagy- and respiratory chain-related proteins (Cox4, Cyc1, NOUFA10, P62, and Pink1) whereas SLC8A3 overexpression could antagonize this trend (Fig. 7D). Additionally, TUNEL staining revealed that SLC8A3 expression counteracted the effects of BRF2 knockout on tumor cell apoptosis in nude mice (Fig. 7E). These findings collectively indicate that BRF2 facilitates tumor progression through its anti-apoptotic function.

## Discussion

Mitochondria-based targeted therapy is more promising than the current combination therapy of programmed death-ligand 1 inhibitors. Although new treatments have improved survival rates, the long-term prognosis of lung cancer remains poor. Therefore, improving the understanding of disease pathogenesis and identifying new molecular targets are crucial. Recently, the use of mitochondria-targeting therapies for targeting cancer stem cells could be valuable [21]. Tumor cell resistance can be overcome by the targeted delivery of anticancer drugs to the mitochondria [22], and cancers resistant to radiotherapy or chemotherapy can be treated, based on nanomaterials, by regulating the redox status [23].

This study demonstrated a link between BRF2 and mitochondrial homeostasis in LUSC, identified and validated the role of *SLC8A3* in LUSC, and linked it to LUSC apoptosis. BRF2 expression was significantly elevated in LUSC tissues and cell lines, which is consistent with the results of previous studies [24]. BRF2 in LUSC may promote tumor progression by inhibiting apoptosis, and animal experiments verified this result. BRF2 inhibits LUAD cell proliferation and metastasis in vivo and in vitro through the MAPK/ERK pathway [25]. Furthermore, BRF2 promotes hepatocellular carcinoma invasion and metastasis through the Wnt/β-catenin pathway [26]. However, the results of this study differ from those of the proliferation, migration, and invasion functions of BRF2 in other tumors. In prostate cancer and breast cancer with high expression of BRF2, pan-active BCL-2 protein family antagonist (Sabutoclax) inhibits tumor progression by promoting cell apoptosis [27, 28]. As apoptosis is a key factor affecting the progression of BRF2-overexpressing LUSC cells, Sabutoclax may be a promising therapeutic option. Unfortunately, this study could not be further explored on this point. The study findings indicate that the downstream target gene, *SLC8A3*, may interact with BRF2 and could have a potential relationship with mitophagy. BRF2 overexpression promoted mitophagy. Immunofluorescence assays confirmed that BRF2 overexpression reduced mitochondrial damage. In eukaryotes, metabolic pathways occur within the cytosol and mitochondria, and glucose or fatty acids provide most of the cellular energy [29]. Mitochondria play a role in energy metabolism and apoptosis [30]. Considering that BRF2 silencing increased apoptosis in LUSC cells, we hypothesized that BRF2 knockdown may affect mitochondrial function in LUSC cells. Gene enrichment results included the AMPK and autophagy-animal signaling pathway. Therefore, BRF2 knockdown possibly affects autophagic activity in LUSC cells. Autophagy is a highly regulated pathway that plays a vital role in regulating primary metabolic functions, enabling cells to remove damaged or harmful components through catabolism and recycling, and maintaining nutrient and energy homeostasis [31]. Autophagy is a central protective mechanism that allows cells to survive in various stress conditions, such as during the production of reactive oxygen species (ROS) [32]. The primary source of intracellular ROS is damaged mitochondria. Therefore, we hypothesized that BRF2-induced apoptosis inhibition in LUSC cells is associated with reduced mitophagy. Mitochondria in cancer cells, which are often characterized by the overproduction of ROS, contribute to cancer development by inducing genomic instability, modifying gene expression, and engaging in signaling pathways [33]. BRF2 overexpression protects cells from apoptosis caused by oxidative stress; however, the link between BRF2 and mitochondria is unknown. The results of this study indicate that mitochondria stabilize with BRF2 upregulation.

The difference between SLC8A3 protein hnRNA levels and mRNA levels increased after BRF2 knockdown. U6 spliceosomal RNA plays an essential role in hnRNA cleavage and is significantly correlated with BRF2 [34]. Therefore, this study suggests that the upregulation of BRF2 expression upregulates *SLC8A3* mRNA at the transcriptional level by regulating *SLC8A3* splicing, which increases SLC8A3 protein expression. SLC8A3 is an integral member of the sodium and calcium exchanger membrane protein family. It mediates the electrical exchange of Ca^2+^ and Na^+^ ions across the cell membrane to regulate cytoplasmic Ca^2+^ levels and Ca^2+^-dependent cellular processes [35–37]. SLC8A3 also contributes to cytosolic Ca^2+^ homeostasis in excitable cells of the muscle, brain, and neurons, helps regulate synaptic plasticity, learning, and memory, mediates mitochondrial Ca^2+^ extravasation and contributes to mitochondrial Ca^2+^ ion homeostasis [38]. However, no studies have confirmed whether SLC8A3 interacts with other proteins to affect the development of lung cancer. Therefore, the molecular mechanisms underlying the role of *SLC8A3* in lung cancer remain unclear. In the classical mitophagy pathway, when mitochondria are damaged, the inner mitochondrial membrane depolarizes, resulting in the accumulation of full-length PINK1 in the outer mitochondrial membrane and the activation of the mitophagy pathway [39]. In this study, SLC8A3 overexpression promoted mitophagy and reversed MMP depolarization and apoptosis in BRF2 knockdown cells. SLC8A3 overexpression counteracts inner mitochondrial membrane depolarization. It contradicts the induction of mitophagy in rescue experiments. Therefore, an unknown mechanism possibly delivers PINK1 to the inner mitochondrial membrane when SLC8A3 is overexpressed. The mitochondrial inner membrane protein TIMM23 is involved in the recognition and transport of proteins encoded by the mitochondrial core. It may be closely related to pink1-mediated mitochondrial autophagy. In this study, SLC8A3 overexpression inhibited PINK1 binding to TIMM23 and induced mitophagy. It reveals a novel pathway of mitophagy.

**Fig. 8:**
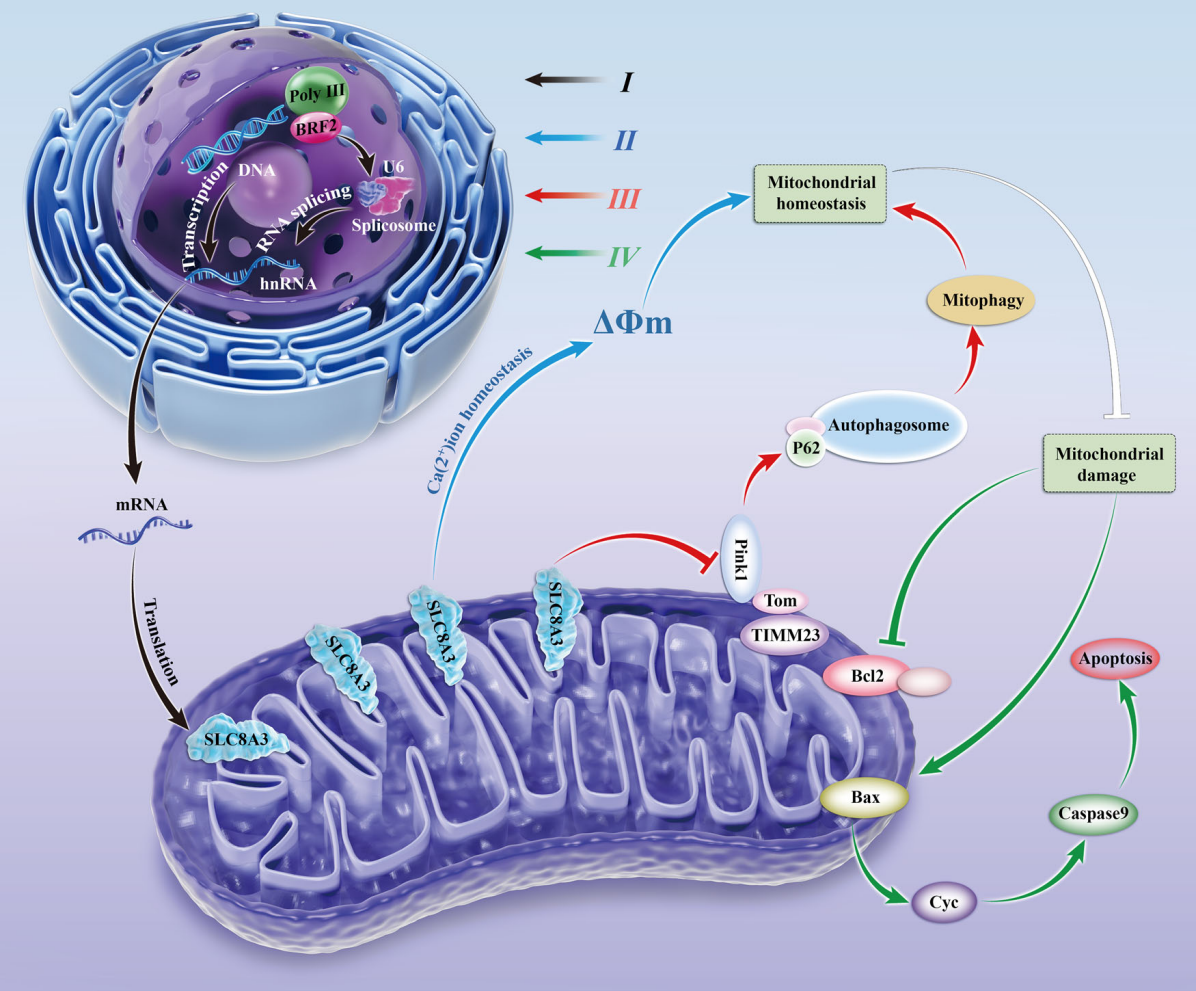
The illustrated mitochondrial autophagy regulatory mechanism of BRF2 in LUSC. (I) BRF2 regulates *SLC8A3* mRNA at the transcriptional level by regulating *SLC8A3* splicing, leading to an increase in SLC8A3 protein expression. (II) SLC8A3 mediates mitochondrial Ca^2+^ and promotes mitochondrial Ca^2+^ ions in the steady state, thereby maintaining MMP. (III) *SLC8A3* overexpression can inhibit the binding of PINK1 to TIMM23, thereby inducing autophagy. (IV) Mitochondrial steady destruction leads to apoptosis.

Therefore, we concluded that BRF2 increased *SLC8A3* expression by regulating the hnRNA splicing of *SLC8A3*. SLC8A3 maintains mitochondrial homeostasis by preserving the MMP and promoting mitophagy. Finally, the damaged mitochondria are reduced to avoid LUSC apoptosis (Fig. 8).

However, this study has certain limitations. The molecular interplay among SLC8A3, PINK1, and TIMM23 remains to be fully elucidated, primarily due to the incomplete understanding of the transport mechanism of PINK1 into the mitochondrial inner membrane. Although BRF2 overexpression could theoretically enhance mitochondrial autophagy, our preliminary in vitro attempts to overexpress BRF2 yielded inconsistent phenotypic outcomes. We hypothesize that a threshold mechanism may exist, potentially limiting its impact on phenotypic functionality. Future investigations should focus on determining whether a linear relationship exists between BRF2 expression levels and mitochondrial membrane potential. Additionally, BRF2 participates in multiple pathways regulating apoptosis in LUSC. The interplay between these distinct mechanisms and their relative contributions to apoptosis regulation warrants further exploration. Consequently, additional studies are essential to dissect these pathways in greater detail and to identify potential therapeutic compounds that could effectively target these molecular players in LUSC treatment.

## Conclusion

This study indicated that BRF2 increases SLC8A3 expression by regulating SLC8A3 hnRNA splicing. SLC8A3 is involved in maintaining mitochondrial homeostasis and reducing damaged mitochondria to prevent LUSC apoptosis. It will be valuable to evaluate the role of SLC8A3 in tumorigenesis in future studies. Furthermore, the finding may elucidate the molecular mechanism of BRF2 in LUSC and provide novel selective therapeutic targets and ideas for LUSC treatment.

## Supplementary information

Supplementary Table 1

Supplementary Table 2

Supplementary Table 3

Western blot

## Data Availability

The data that support the findings of this study are accessible from the corresponding author upon reasonable request.
