# Supplementary material for: TFIIB-related factor 2 inhibits lung squamous carcinoma cell apoptosis through SLC8A3-mediated mitochondrial homeostasis
Source: Cell Death Dis. 2025 Jul 3;16(1):491. doi: 10.1038/s41419-025-07813-8 (PMC12229314; doi:10.1038/s41419-025-07813-8)
Supplement: Supplementary file 1 — Supplementary Table 1 [file 41419_2025_7813_MOESM1_ESM.docx]

**Supplementary Table 1 Sequence of small interfering RNA**

| SiRNA sequence | sense（5'-3'） | antisense（5'-3'） |
| --- | --- | --- |
| Scramble | UUCUCCGAACGUGUCACGUTT | ACGUGACACGUUCGGAGAATT |
| BRF2-Si1 | CAGCCAAAUACGUGGAAGATT | UCUUCCACGUAUUUGGCUGTT |
| BRF2-Si2 | GCACUUACAUGCAGAUAGUTT | ACUAUCUGCAUGUAAGUGCTT |
| TIM23-Si1 | GCAUGAAGCUCAUCGACUATT | UAGUCGAUGAGCUUCATGCTT |
| TIM23-Si2 | CCUACGUCAUCGAGUACAATT | UUGUACUCGAUGACGUAGGTT |
| TIM23 | UUCUCCGAACGUGUCACGUTT | ACGUGACACGUUCGGAGAATT |
