# Supplementary material for: TFIIB-related factor 2 inhibits lung squamous carcinoma cell apoptosis through SLC8A3-mediated mitochondrial homeostasis
Source: Cell Death Dis. 2025 Jul 3;16(1):491. doi: 10.1038/s41419-025-07813-8 (PMC12229314; doi:10.1038/s41419-025-07813-8)
Supplement: Supplementary file 2 — Supplementary Table 2 [file 41419_2025_7813_MOESM2_ESM.docx]

**Supplementary Table 2 Information of antibodies**

| BRF2 | A8782 | 1:1000 | abclonal, China |
| --- | --- | --- | --- |
| β-Tubulin | EM0103 | 1:5000 | HUABIO, China |
| SLC8A3 | bx027025 | 1:1000 | Abbexa, UK |
| COX4 | AF5468 | 1:1000 | Affinity, China |
| CYC1 | DF4698 | 1:1000 | Affinity, China |
| NDUFA10 | DF2211 | 1:1000 | Affinity, China |
| P62 | 80294-1-RR | 1:2000 | Proteintech, China |
| PINK1 | 6946 | 1:1000 | Cell Signaling Technology, USA |
| BCL2 | ET1702-53 | 1:1000 | HUABIO, China |
| BAX | 50599-2-IG | 1:2000 | Proteintech, China |
| CASPASE9 | 10380-1-AP | 1:1000 | Proteintech, China |
| HA | C29F4 |  | Cell Signaling Technology, USA |
| FLAG*3 | Ae092 |  | abclonal, China |
| TIM23 | 67535-1-IG | 1:1000 | Proteintech, China |
