# Supplementary material for: TFIIB-related factor 2 inhibits lung squamous carcinoma cell apoptosis through SLC8A3-mediated mitochondrial homeostasis
Source: Cell Death Dis. 2025 Jul 3;16(1):491. doi: 10.1038/s41419-025-07813-8 (PMC12229314; doi:10.1038/s41419-025-07813-8)
Supplement: Supplementary file 3 — Supplementary Table 3 [file 41419_2025_7813_MOESM3_ESM.docx]

**Supplementary Table 3 Sequence of Primer**

| Primer Sequence(5’→3’) | Forward（5'-3'） | Reverse（5'-3'） |
| --- | --- | --- |
| Primer 1 | CTGCTTGCCTTCCTGCCC | GAAACCCCGACCCAGAGC |
| Primer 2 | CACCACGCTCTTGCTTCCTA | CTCCAAAAACCCAGGCTCCA |
