# Supplementary material for: TFIIB-related factor 2 inhibits lung squamous carcinoma cell apoptosis through SLC8A3-mediated mitochondrial homeostasis
Source: Cell Death Dis. 2025 Jul 3;16(1):491. doi: 10.1038/s41419-025-07813-8 (PMC12229314; doi:10.1038/s41419-025-07813-8)
Supplement: Supplementary file 4 — Western blot [file 41419_2025_7813_MOESM4_ESM.pdf]

# F1

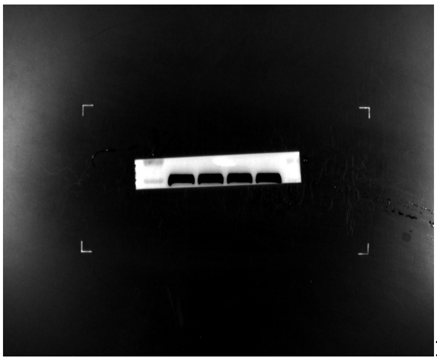

1B-TUBULIN

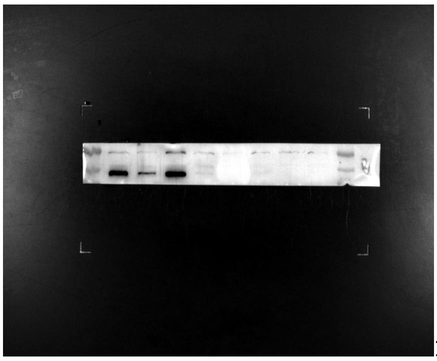

1-BRF2

# F2

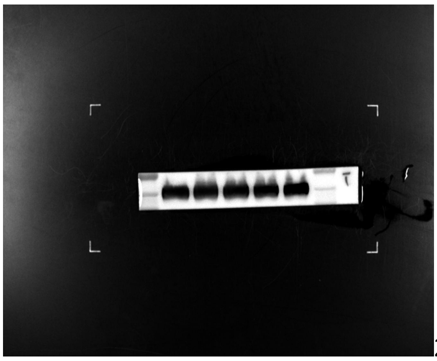

2A-TUBULIN

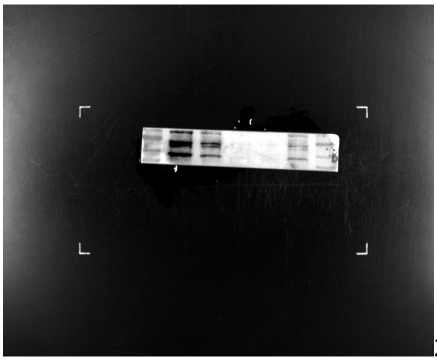

2A-H226 -BRF2

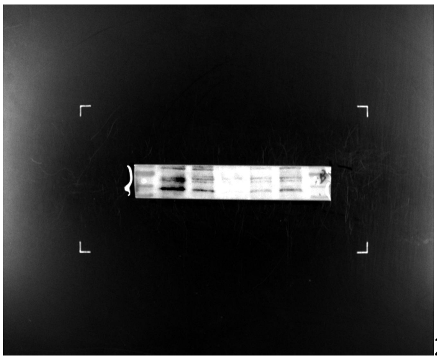
2A-H520-BRF2

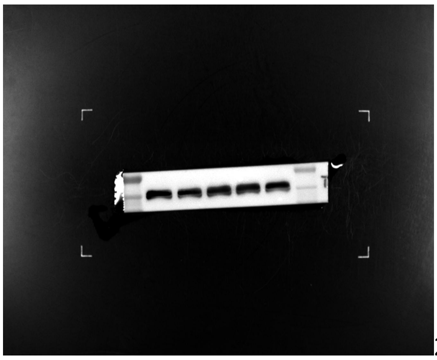
2A-H520-TUBULIN

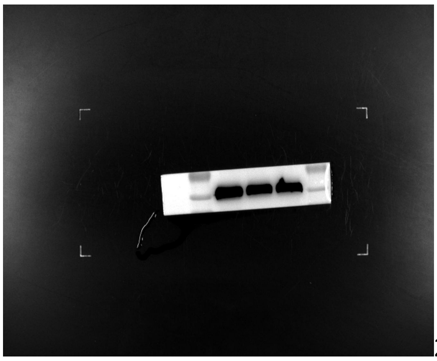
2C-H226-TUBULIN

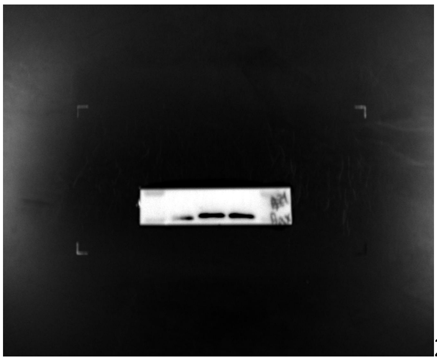
2C-H226-BAX

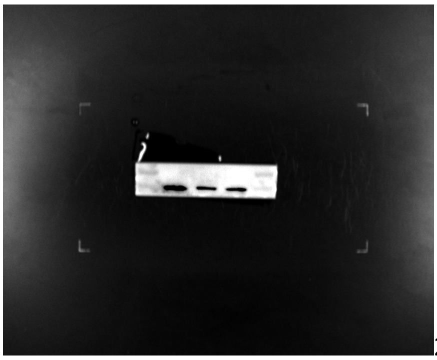
2C-H226-BCL

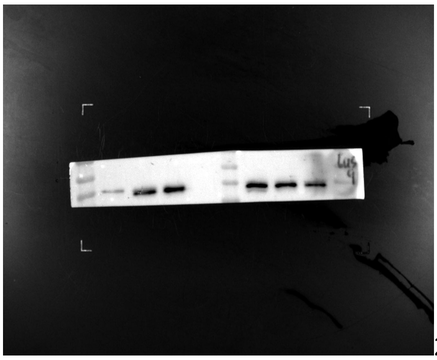
2C-H226-C-cas9

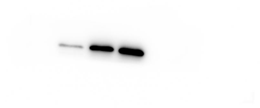
2C-H520-BAX

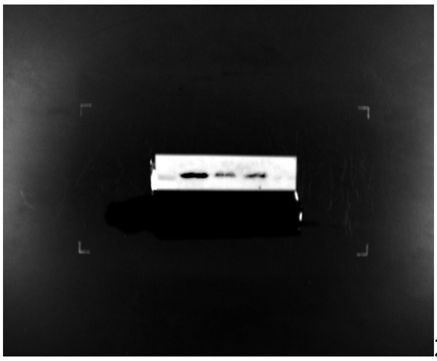
2C-H520-BCL

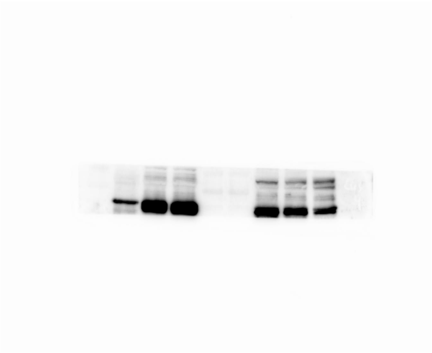
2C-H520-C-cas9

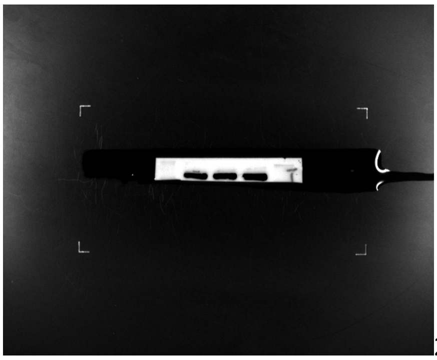
2C-H520-TUBULIN

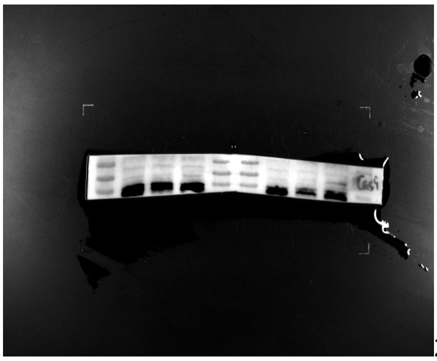

2C-H226-cas9

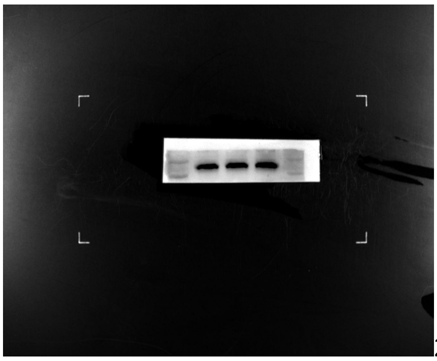
2C-H520-cas9

# F3

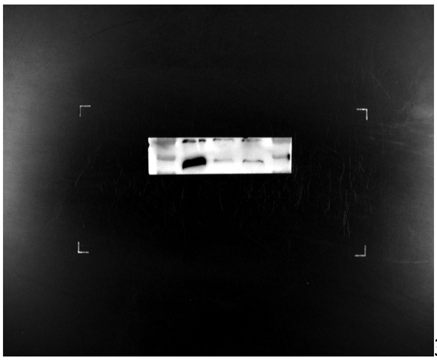
3D-H226-BRF2

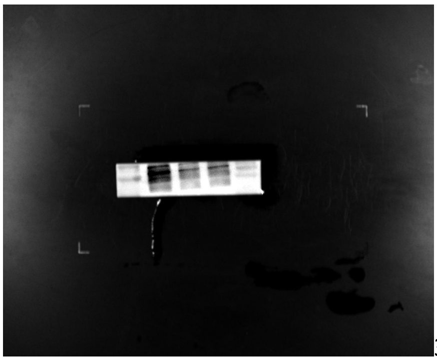
3D-H226-SLC8A3

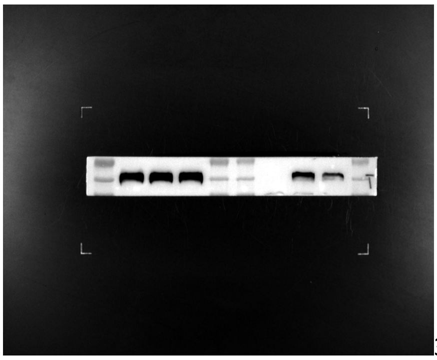
3D-H226-TUBULIN

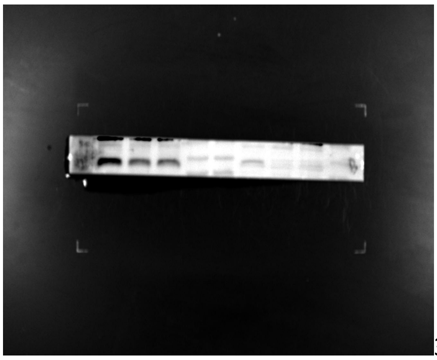
3D-H520-BRF2

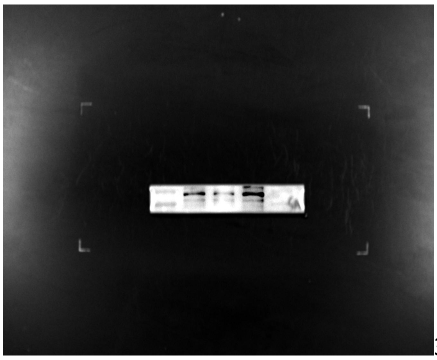
3D-H520-SLC8A3

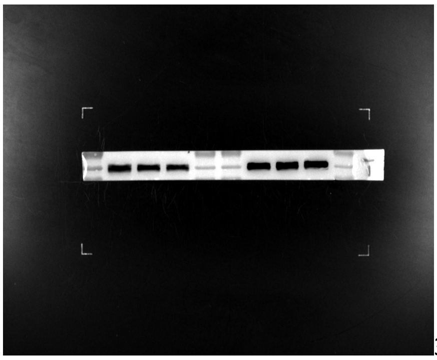
3D-H520-TUBULIN

# F4

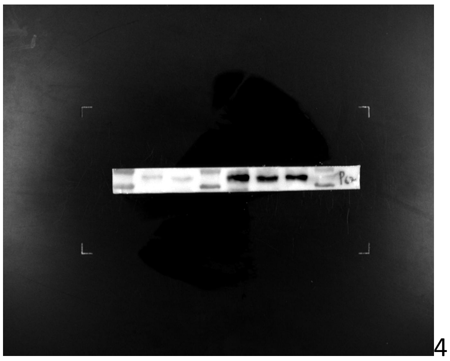
4B-H226-p62

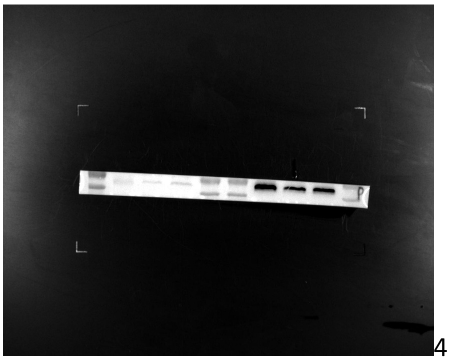
4B-H226-PINK1

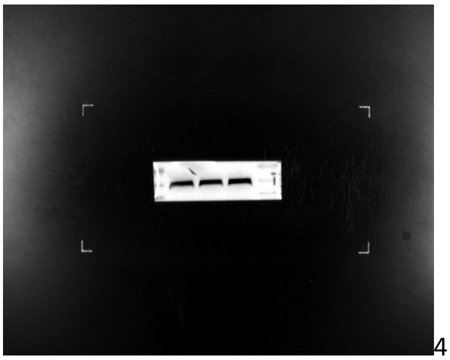
4B-H226-TUBULIN

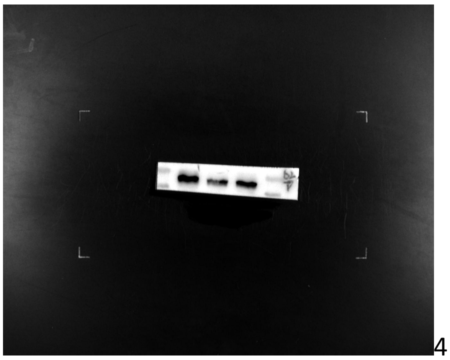
4B-H520-p62

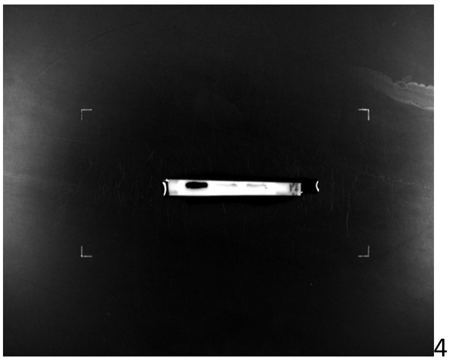
4B-H520-PINK1

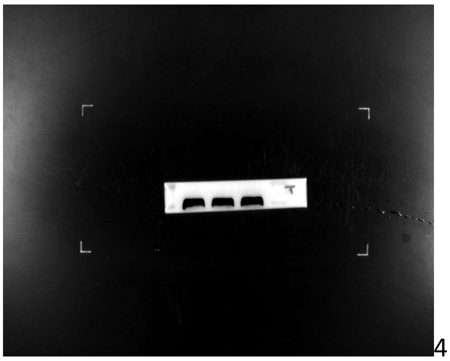
4B-520-TUBULIN

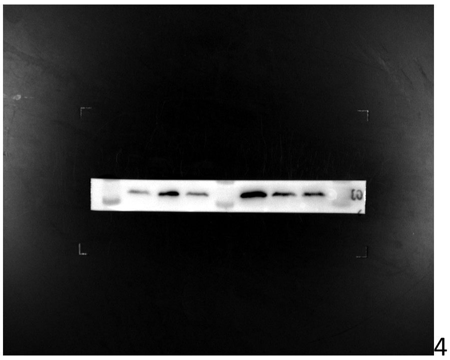
4D-H226-COX4

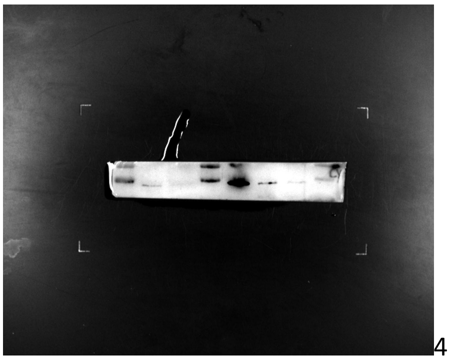
4D-H226-CYC1

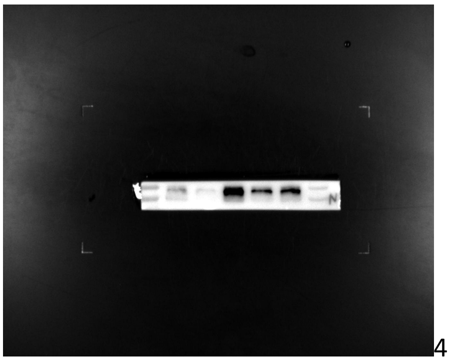
4D-H226-NDUFA10

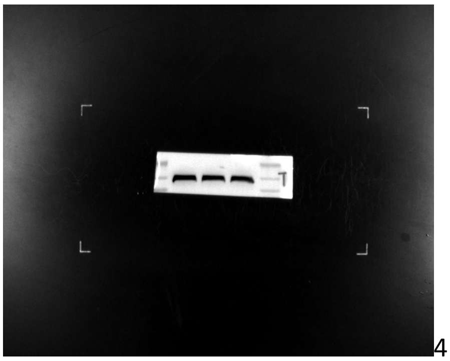
4D-H226-TUBULIN

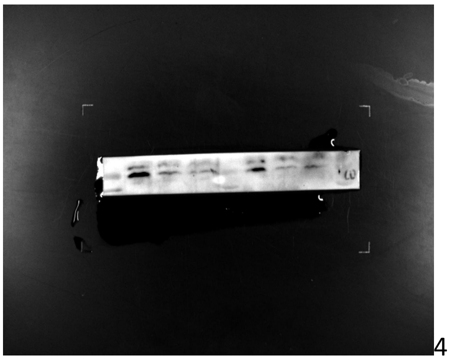
4D-H520-COX4

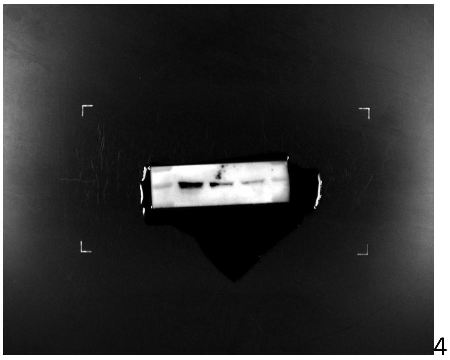
4D-H520-CYC1

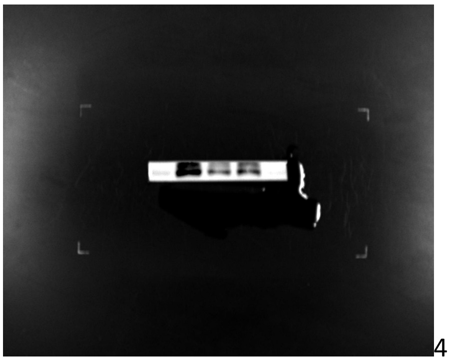
4D-H520-NDUFA10

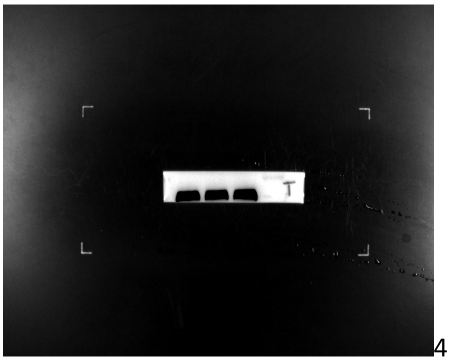
4D-H520-TUBULIN

# F5

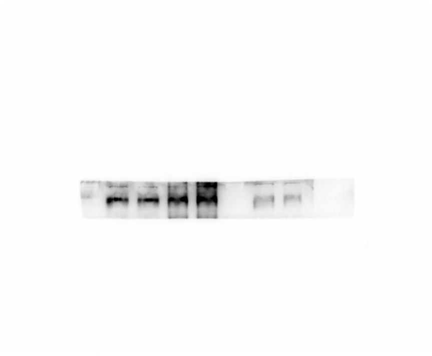

NC-SLC8A3

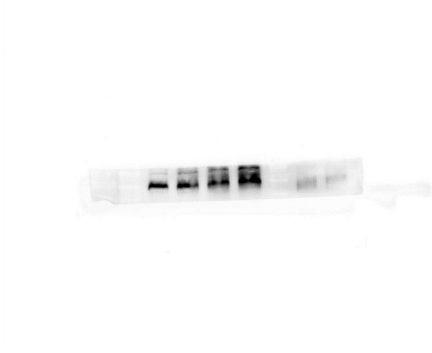

OE-SLC8A3

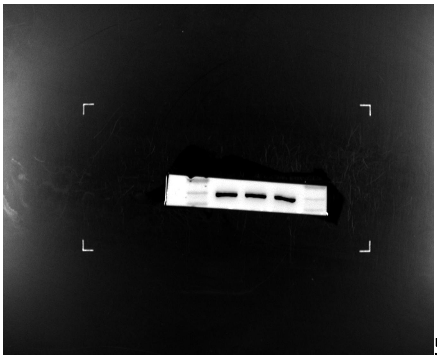

5B-293T-TUBULIN

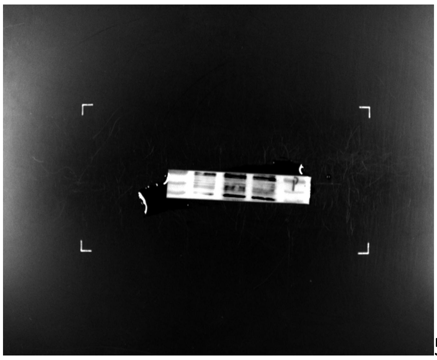

5B-293T-PINK1

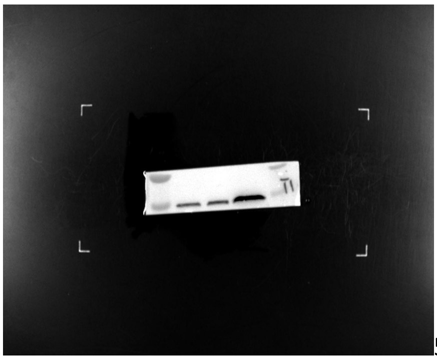
5B-TIM23

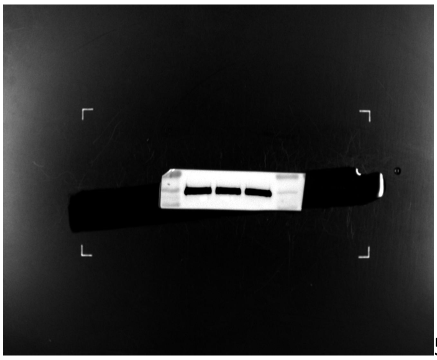
5C-H226-TUBULIN

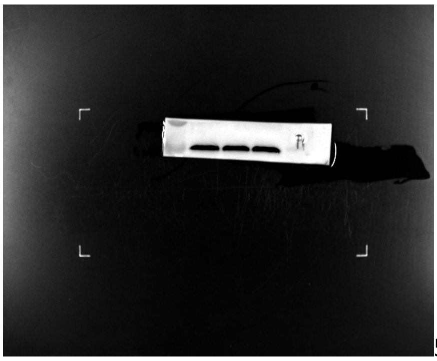
5C-H520-TIM23

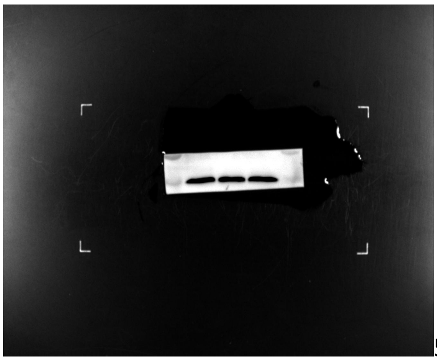
5C-H226-TIM23

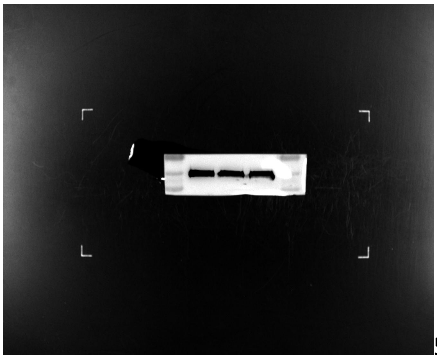

5C-H226-TUBULIN

# F6

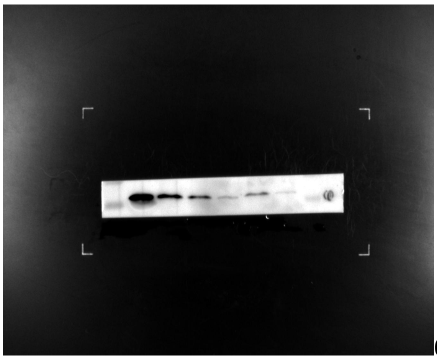

6A-H226-COX4

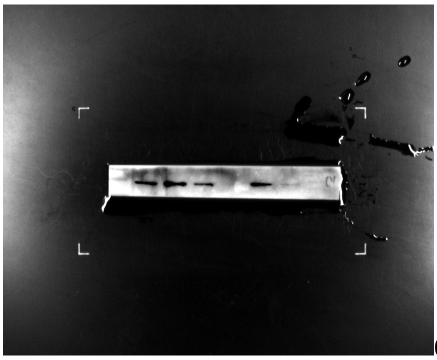

6A-H226-CYC1

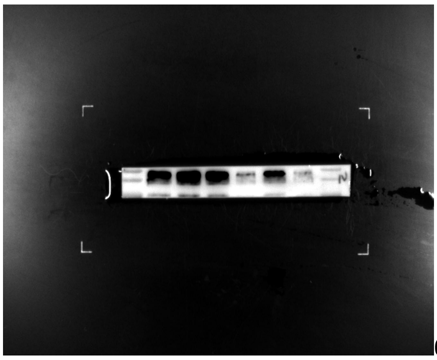

6A-H226-NDUFA10

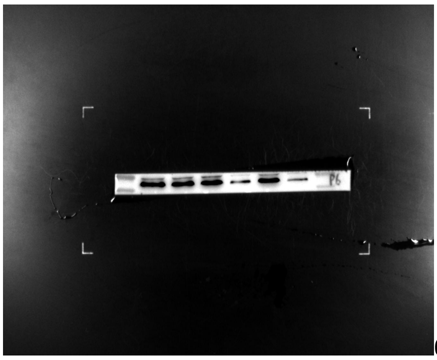
6A-H226-p62

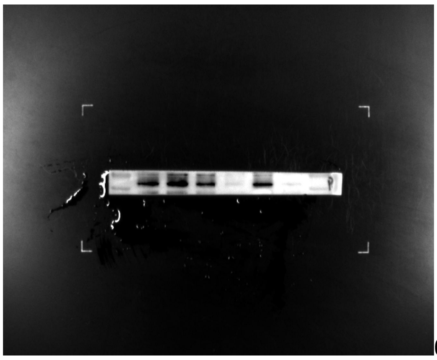
6A-H226-PINK1

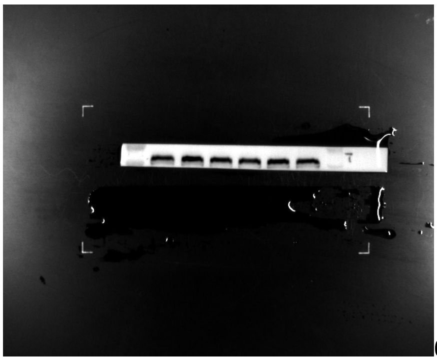
6A-H226-TUBULIN

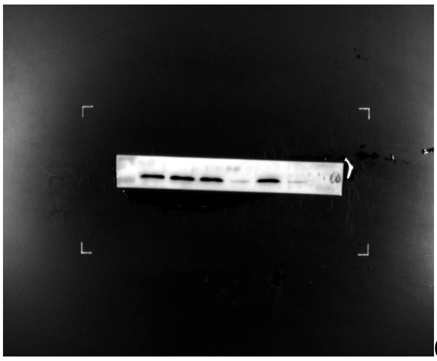
6A-H520-COX4

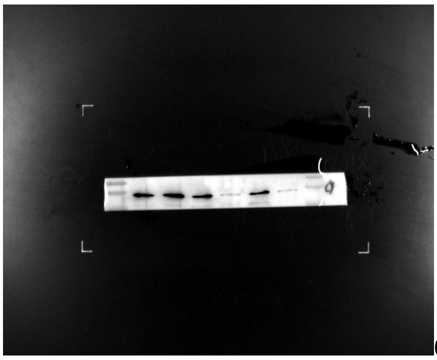
6A-H520-CYC1

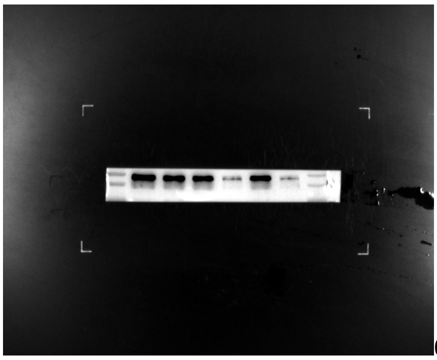
6A-H520-NDUFA10

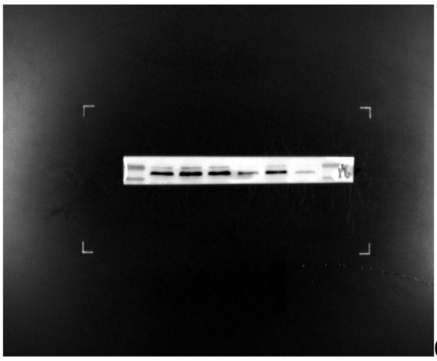
6A-H520-p62

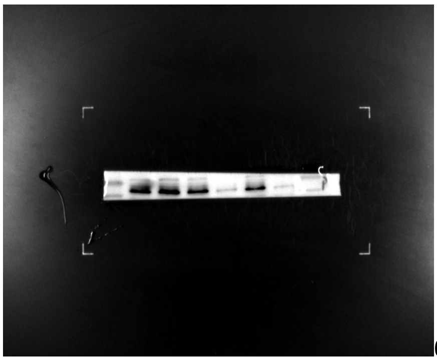
6A-H520-PINK1

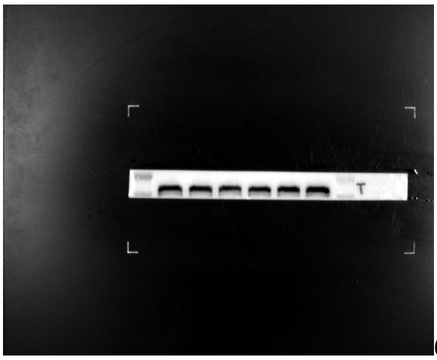
6A-H520-TUBULIN

# F7

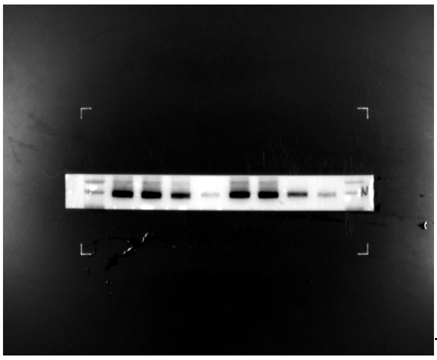
7D-H226-NDUFA10

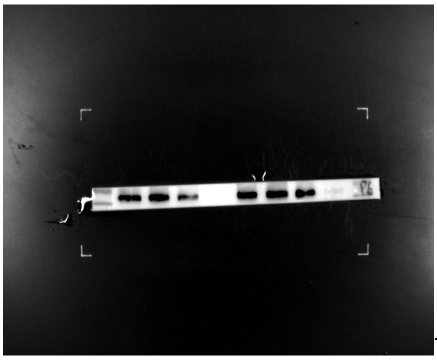
7D-p62

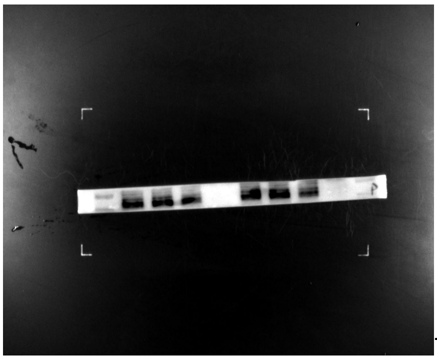
7D-H226-PINK1

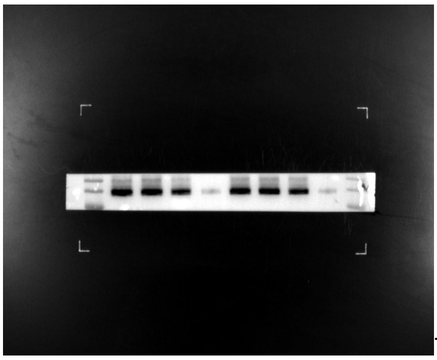
7D-H520-NDUFA10

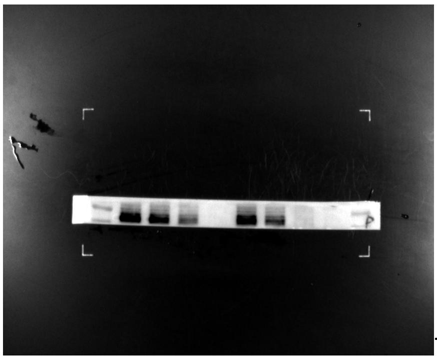
7D-H520-PINK1

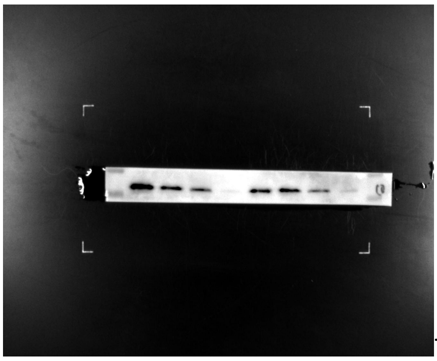
7D-COX4

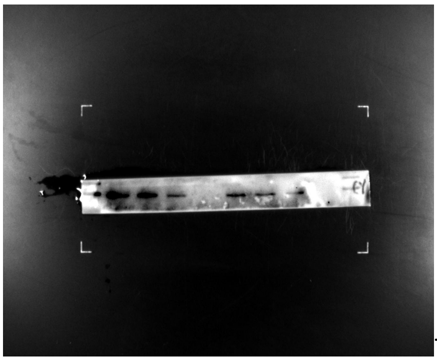
7D-CYC1

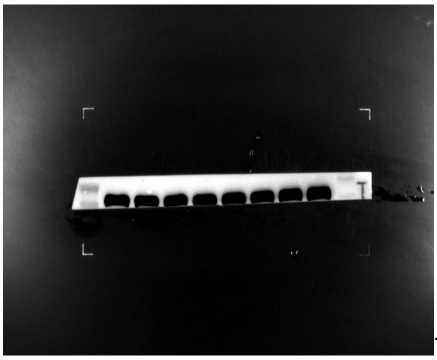
7D-TUBULIN
